# Advancements in Neuroimaging to Unravel Biological and Molecular Features of Brain Tumors

**DOI:** 10.3390/cancers13030424

**Published:** 2021-01-23

**Authors:** Francesco Sanvito, Antonella Castellano, Andrea Falini

**Affiliations:** 1 Neuroradiology Unit and CERMAC, IRCCS Ospedale San Raffaele, 20132 Milan, Italy; francesco.sanvito01@universitadipavia.it (F.S.); falini.andrea@hsr.it (A.F.); 2 School of Medicine, Vita-Salute San Raffaele University, 20132 Milan, Italy; 3 Unit of Radiology, Department of Clinical, Surgical, Diagnostic, and Pediatric Sciences, University of Pavia, 27100 Pavia, Italy

**Keywords:** brain tumors, advanced MRI, molecular profiling, perfusion-weighted imaging, diffusion MRI, magnetic resonance spectroscopy, radiomics, radiogenomics, quantitative imaging

## Abstract

**Simple Summary:**

Advanced neuroimaging is gaining increasing relevance for the characterization and the molecular profiling of brain tumor tissue. On one hand, for some tumor types, the most widespread advanced techniques, investigating diffusion and perfusion features, have been proven clinically feasible and rather robust for diagnosis and prognosis stratification. In addition, 2-hydroxyglutarate spectroscopy, for the first time, offers the possibility to directly measure a crucial molecular marker. On the other hand, numerous innovative approaches have been explored for a refined evaluation of tumor microenvironments, particularly assessing microstructural and microvascular properties, and the potential applications of these techniques are vast and still to be fully explored.

**Abstract:**

In recent years, the clinical assessment of primary brain tumors has been increasingly dependent on advanced magnetic resonance imaging (MRI) techniques in order to infer tumor pathophysiological characteristics, such as hemodynamics, metabolism, and microstructure. Quantitative radiomic data extracted from advanced MRI have risen as potential in vivo noninvasive biomarkers for predicting tumor grades and molecular subtypes, opening the era of “molecular imaging” and radiogenomics. This review presents the most relevant advancements in quantitative neuroimaging of advanced MRI techniques, by means of radiomics analysis, applied to primary brain tumors, including lower-grade glioma and glioblastoma, with a special focus on peculiar oncologic entities of current interest. Novel findings from diffusion MRI (dMRI), perfusion-weighted imaging (PWI), and MR spectroscopy (MRS) are hereby sifted in order to evaluate the role of quantitative imaging in neuro-oncology as a tool for predicting molecular profiles, stratifying prognosis, and characterizing tumor tissue microenvironments. Furthermore, innovative technological approaches are briefly addressed, including artificial intelligence contributions and ultra-high-field imaging new techniques. Lastly, after providing an overview of the advancements, we illustrate current clinical applications and future perspectives.

## 1. Introduction

The 2016 World Health Organization (WHO) classification of central nervous system (CNS) tumors [1] introduced molecular characterization as a crucial step in the diagnosis of brain neoplasms, which are categorized both in grades reflecting their microscopical features and in molecular subtypes. Infiltrating lower-grade gliomas (LGG, grade 2/3), i.e., astrocyte- and oligodendrocyte-derived tumors, currently include isocytrate dehydrogenase (IDH) mutant 1p/19q-codeleted (IDH^mut^1p19q^cod^) oligodendroglioma, IDH-mutant 1p/19q-intact (IDH^mut^1p19q^int^) astrocytoma, and IDH-wildtype (IDH^wt^) astrocytoma. Similarly, glioblastomas (GBM, grade 4) acknowledge IDH^mut^ and IDH^wt^ subtypes. The molecular characterization of these tumors is crucial in the diagnostic workup, as the molecular profile outweighs tumor grade for risk stratification [2], LGG-IDH^wt^ showing a prognosis almost similar to GBM-IDH^wt^, with GBM-IDH^mut^ being less aggressive. Medulloblastomas, malignant embryonal tumors (grade 4) relatively common in the pediatric population and arising in the posterior fossa, also acknowledge new molecular subtypes, depending on WNT- and Sonic Hedgehog (SHH-)status [3]. In addition, the 2016 classification introduced diffuse midline gliomas H3K27M-mutated (DMG-H3K27M^mut^, grade 4), a newly defined tumor type characterized by worse prognosis [4] than the other tumors located in similar regions—mostly thalamus or posterior fossa.

Considering the limited possibility and the invasiveness of acquiring tissue samples for histopathological evaluations and genetic profiling, the clinical assessment would dramatically benefit from noninvasive in vivo biomarkers predicting tumor grades and molecular subtypes at the time of diagnosis and reflecting biological changes over time. In recent years, magnetic resonance imaging (MRI) has been increasingly employed for the extraction of quantitative data reflecting underlying pathophysiological characteristics of the neoplastic tissue—a method named “radiomics” [5,6,7].

Quantitative imaging-derived features for radiomic analyses can be extracted from either conventional MRI (cMRI) or advanced MRI (aMRI, including diffusion MRI (dMRI) and perfusion-weighted imaging (PWI)) maps, by using three-dimensional (3D) volumes of interest (3D-VOIs) containing the whole tumor or two-dimensional (2D) in-plane regions of interest (2D-ROIs) corresponding to “virtual samples” of tumor tissue. Radiomic features include shape features describing the geometric appearance of the tumor, histogram-derived statistics (“first-order” features) providing information about the distribution of voxel intensities within the tumor (e.g., mean, median, percentiles, skewness), and textural features (“second-order” features) quantifying the spatial patterns of voxel intensities among adjacent voxels [8,9].

As a “rule of thumb”, molecular profiles bearing a worse prognosis exhibit more “aggressive” aMRI markers [10,11,12], such as lower apparent diffusion coefficient (ADC)—reflecting higher cellularity and extracellular matrix alterations [13]—and higher perfusion/permeability parameters—corresponding to microvascular proliferation and leakage [10,11,12,13,14]. More in detail, dMRI features multiple techniques providing parameters that quantify water displacement in the extracellular space (ADC), that estimate the preferential orientation of water motion (diffusion tensor imaging—DTI, and diffusion kurtosis imaging—DKI) [15,16], and that quantify the reciprocal representation of different water compartments (biophysical models) [17]. On the other hand, PWI includes three main techniques (dynamic susceptibility contrast—DSC, dynamic contrast-enhanced—DCE, and arterial spin labeling—ASL) that are variously based on exogenous (DSC, DCE) or endogenous (ASL) tracers, and that provide insightful metrics reflecting tissue perfusion/permeability: blood volume (DSC-cerebral blood volume (CBV)), blood flow (DSC- and ASL-cerebral blood flow (CBF)), intravascular tracer (DCE-V_p_), and parameters describing the tracer exchange between intravascular and extracellular space (DCE-K^trans^, -V_e_, -K_ep_) [14,18]. While dMRI and PWI infer tumor pathophysiological features by assessing microstructural and perfusion/permeability tissue properties, novel magnetic resonance spectroscopy (MRS) approaches enable the detection of specific diagnostic molecules, as in the case of 2-hydroxyglutarate (2HG), which accumulates in brain tumors as a result of IDH-mutation [19], thus representing the first instance of MRI directly measuring a mutation marker in brain tumors.

In the first part of this review, we discuss the diagnostic performance of quantitative data derived from PWI, ADC, and MRS in the molecular profiling of brain tumors, including recently defined peculiar tumor entities. In the second part, we present novel and promising aMRI approaches (DTI/DKI, biophysical models, tumor blood-oxygen-level-dependent imaging (BOLD)) that may provide deeper insights regarding tumor extension, microstructure, and microvasculature. In the third part, we briefly address new approaches based on ultra-high-field imaging and contributions from artificial intelligence. Lastly, we conclude by discussing the clinical applicability and future directions.

## 2. IDH-Status Prediction in Gliomas through Perfusion and Diffusion Assessment

For glial tumors, isocytrate dehydrogenase (IDH) status is currently the most important molecular feature to assess in gliomas, as the absence of IDH-mutation dramatically worsens prognosis both in LGG and in GBM [2]. IDH-mutation is considered an early event in gliomagenesis, and it results in the overproduction of 2HG, an oncometabolite influencing cellular metabolism, genetic stability, and epigenetic phenomena [20]. IDH-determination is a fundamental step in the diagnostic workup, as therapeutic decisions such as the timing of radiation-treatment are influenced by IDH-status [21], and it is also becoming of crucial importance to identify patients that may benefit from IDH-targeted therapy [22]. A recent meta-analysis [23] listed the MRI hallmarks of IDH^mut^-gliomas: preferential frontal lobe location, lower probability of contrast enhancement, well-defined borders, T2-FLAIR (fluid-attenuated inversion recovery) mismatch (T2FM) sign, higher ADC, lower fractional anisotropy (FA), and lower cerebral blood volume (CBV)—with high summary sensitivity/specificity (0.86/0.87; area under the curve (AUC), 0.93) of MRI for IDH-prediction. Multiple studies have assessed the diagnostic performance of aMRI for IDH-status prediction, mainly on the basis of dMRI, PWI, or combination approaches.

Multiple studies assessed the capability of PWI for IDH-status determination in LGG, with relative-CBV (rCBV) being the parameter most frequently employed. In a cohort of 73 treatment-naïve LGG, Kickingereder and colleagues [24] demonstrated that IDH^mut^-LGG were characterized by a decreased hypoxia-induced angiogenesis, as well as reduced rCBV histogram metrics, and they reported an excellent performance of 90th percentile-rCBV (AUC 0.92, PPV/NPV 0.89/078) for IDH-mutation prediction. Lee and colleagues [25] also employed histogram-derived rCBV-metrics and found rCBV-skewness being a significant predictor of IDH^mut^1p19q^int^-genotype among LGG (AUC 0.69). In another study enrolling LGG-patients [26], an ROI-derived measure (rCBV_max_, comparing tumor-CBV to the contralateral white matter) also exhibited a good diagnostic performance (AUC 0.82), and the authors proposed a threshold of <2.35 for high-sensitivity IDH-status prediction (sensitivity/specificity 1.00/0.61 in their cohort), meaning that rCBV_max_ > 2.35 is highly indicative of IDH^wt^-status and, therefore, of worse prognosis.

In other articles, various measures derived from ADC-maps, including histogram-derived metrics (e.g., mean, median, percentiles, skewness, entropy) and ROI-derived values (e.g., mean, minimum, ratios), have been applied for the prediction of IDH-status specifically on LGG. Across studies [25,26,27,28], 2D-ROI measures seemed to perform slightly better than histogram-derived ones (AUC ranging 0.83–0.96 vs. 0.75–0.94, respectively), and a 1.8 threshold for the ratio between the mean single-slice ADC and the normal-appearing white matter (ADC_mean_/ADC_NAWM_) is to be considered the most reliable diffusion-metric for IDH-status, as it demonstrated good diagnostic performance in two distinct studies [27,28] (sensitivity/specificity: ~0.83/~0.95 and 0.87/0.60, respectively). These studies, particularly the one by Thust et al. [28], have relevant clinical implications, demonstrating high accuracy of ADC-measurements manually performed with 2D-ROIs and, therefore, applicable in the clinical routine. In addition, moving from the evidence that IDH^mut^1p19q^int^-LGG are the only LGG expected to contain large subvolumes with high ADC-values, other authors [29] proposed an alternative index for their identification: the percentage of tumor volume with ADC > 1.5 × 10^−3^ mm^2^/s (V_ADC>1.5_), which also demonstrated good accuracy (~0.8 AUC in both their training and validation cohort).

**Figure 1:**
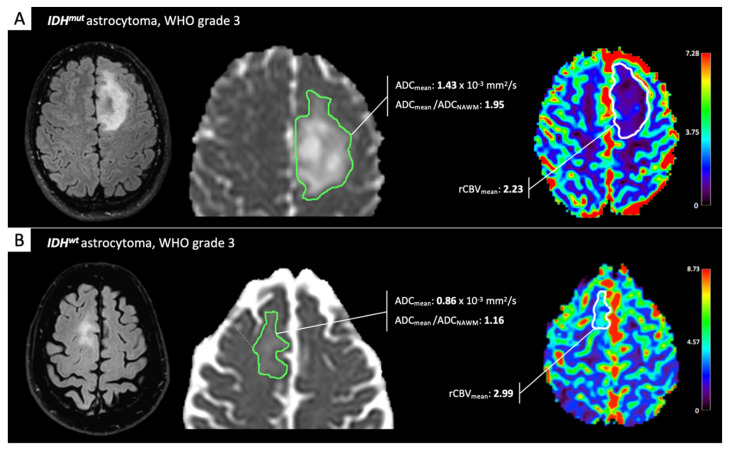
Perfusion and diffusion assessment of lower-grade glioma (LGG). MRI datasets were retrieved from the archive of our Institution. Diffusion MRI (dMRI) and perfusion-weighted imaging (PWI) features from one isocitrate dehydrogenase mutant (IDH^mut^)-LGG (**A**) and one IDH wild type (IDH^wt^)-LGG (**B**) were evaluated through a clinically feasible single-slice region of interest (ROI) approach. IDH^wt^-LGG exhibits more “aggressive” aMRI features, in particular a mean apparent diffusion coefficient (ADC_mean_)/normal-appearing white matter (ADC_NAWM_) ratio lower than 1.8, the proposed cutoff for IDH^wt^ status prediction by Maynard et al. [27] and Thust et al. [28].

The aforementioned studies, taken together, seem to advocate for a rather high reliability of ADC and PWI for IDH-status prediction in LGG. However, many authors [11,25,26,27] demonstrated that the diagnostic performance of aMRI for IDH-status in LGG significantly increases when combined in multimodality analyses and/or with cMRI features. ADC- and/or rCBV-measures have been variously combined with cMRI features—such as tumor volume, enhancement, and location, and presence of calcifications, cysts, or T2FM—with very good to excellent diagnostic performance for IDH-determination (AUC ranging 0.84–0.96 across studies). Maynard and colleagues [27], for instance, obtained strong results on a large LGG cohort (study/test sample: 290/49 patients), by developing two models based on ROI-derived ADC_mean_/ADC_NAWM_ combined with the patient’s age, tumor location and enhancement pattern, and presence of calcifications (model-A, AUC 0.96) or cysts (model-B, AUC 0.94). Figure 1 shows two representative cases (one IDH^mut^- and one IDH^wt^-LGG, respectively) for which diffusion and perfusion features were evaluated through a clinically feasible single-slice ROI approach.

Similarly to LGG, quantitative-aMRI has also been employed for IDH-prediction in cohorts including GBM. Zhang and colleagues [30] explored several histogram-derived PWI-metrics and identified 10th percentile AUC (area under the curve, a DCE-metric) (sensitivity/specificity/AUC 0.78/0.80/0.83) and 10th percentile-K^trans^ (sensitivity/specificity/AUC 0.78/0.85/0.80) as the best predictors of IDH-status in a cohort including both LGG and GBM. Other authors proposed cerebral blood flow (CBF) derived from ASL as an IDH-predictor in cohorts including exclusively GBM [31] or both LGG and GBM [32]. The former article used ROI-derived CBF_max_ from the enhancing tissue, with sensitivity/specificity 0.58/0.79; the latter employed VOI-extracted whole-tumor CBF, with higher sensitivity/specificity 0.77/0.88, and reported ASL-CBF superiority to other PWI-metrics. The combined usage of aMRI markers seems to lead to better results also in GBM. A recent study [33], for instance, compared ROI-derived tumoral-ADC_min_, tumoral-CBV_max_, and peritumoral-CBV_max_ for IDH-prediction; the multimodal combination of several aMRI markers improved the diagnostic performance (AUC 0.88–0.9 for combined approaches vs. 0.7–0.89). Furthermore, consistently with “aggressive” aMRI markers usually reflecting a worse prognosis, findings by Wu and colleagues [12] not only confirm lower ADC-values in IDH^wt^-gliomas (including both LGG and GBM), but also suggest that, to some extent, ADC may reflect tumor “aggressiveness” even regardless of genotyping; in their cohort, IDH^mut^-gliomas exhibiting low ADC-values (rADC_mean_ < 1.08) presented a prognosis comparable to IDH^wt^-gliomas.

Overall, CBV- and ADC-based measurements are the most robust aMRI markers. However, while ADC ROI-measurements were proven rather reproducible between scanners and at different field strengths [34,35], the comparison of CBV-estimates (and cutoffs) across different centers still suffers from a number of confounding variables. In particular, different institutions apply various countermeasures for leakage-effects—including preload (at different dosages), lowering the flip angle, and/or post-processing leakage correction—that may contribute to variability in CBV-estimation. This limitation of DSC is well recognized, and there have been increasing efforts to move toward more standardized protocols [36,37,38]. Furthermore, achieving a definitive multicentric consensus on DSC would potentially enable the inclusion of PWI in the treatment response assessment for clinical trials, which is currently based exclusively on cMRI findings and measurements, according to 2010 Response Assessment in Neuro-Oncology (RANO) criteria [39]. In fact, rCBV is currently the most validated aMRI-metric for distinguishing therapy effects such as pseudoprogression or radiation-induced injury from progressive disease [36,38], and DSC standardization would contribute to reduce the current variability in the proposed cutoffs for differential diagnosis [40]. Conversely, although lower ADC values have been associated with progressive disease compared to pseudoprogression, the use of ADC alone is still controversial for this distinction due to the intrinsic glioma heterogeneity, with regions of high cellularity mixed with areas of necrosis, edema, and microhemorrhage that can be found both in pseudoprogression and in radiation-induced injury [40]. On the other hand, the inclusion of diffusion-weighted imaging (DWI) in the response assessment of antiangiogenic agents, such as bevacizumab, may improve the evaluation of therapy effects such as pseudoresponse, in which a decrease in the enhancing area with progressively growing restricted diffusion lesion burden is associated with a worse prognosis [40].

## 3. Spectroscopy Advancements: 2-Hydroxyglutarate Direct Detection to Demonstrate IDH-Mutation

In recent years, 2HG-MRS allowed the measurement of 2HG accumulation as a result of IDH-mutation, thus representing the first instance of MRI enabling the direct detection of a molecular marker in neuro-oncology. Since the first pulsed sequence for 2HG measurement was optimized [19], this approach has been explored for IDH-status prediction at time of diagnosis and for monitoring 2HG levels over time. According to a recent meta-analysis by Suh and colleagues [41], across 14 studies, 2HG-MRS consistently measured higher 2HG-accumulation in IDH^mut^-gliomas, with high diagnostic performance for IDH-determination (summary AUC 0.96; pooled sensitivity/specificity 0.95/0.91). 2HG-peak was evaluated at 2.25 ppm, in the majority of cases, and cutoff values for 2HG-peak varied from 0.897 to 2 mM. For instance, two studies [42,43] proposed thresholds of 1 mM and 2 mM (respectively) for the identification of the 2HG-peak. Grouping individual patients’ data provided by five out of the 14 studies included (173 patients), Suh et al. [41] identified 1.76 mM as the optimal cutoff value for IDH-determination, with sensitivity/specificity 0.75/0.94.

In addition to its diagnostic contribution, a potential role in treatment response assessment has been proposed for of 2HG-MRS, as 2HG-levels decrease following cytotoxic treatment [42,44] and increase sharply in case of tumor progression [42] in IDH^mut^-gliomas. This application may be particularly relevant in the case of IDH-targeted drugs, such as ivosidenib and vorasidenib [21], as 2HG-MRS would represent a unique tool for directly measuring the drug-induced inhibition of IDH activity. Andronesi et al. [45] longitudinally evaluated patients diagnosed with IDH1^mut^-gliomas and treated with IDH305 inhibitor, by measuring 2HG concentration in tumor tissue pre and post treatment. 2HG-MRS demonstrated a rapid decrease of 2HG levels (70% decrease in 2HG/hCr ratio) after 1 week of IDH1-inhibition.

**Figure 2:**
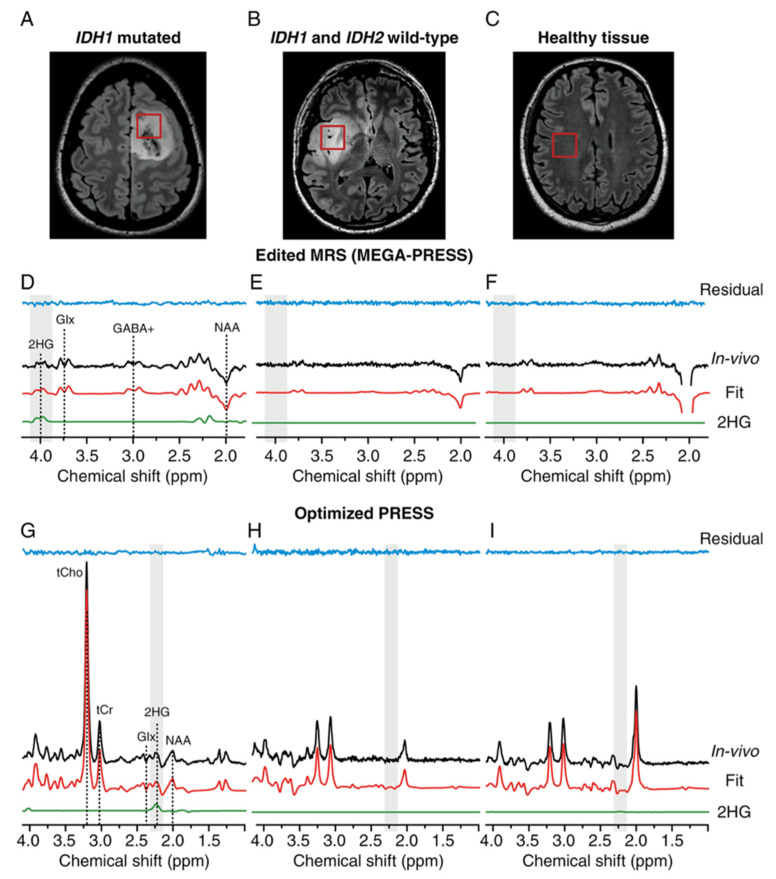
2-Hydroxyglutarate (2HG) magnetic resonance spectroscopy (MRS) of one IDH^mut^-glioma (first column), one IDH^wt^-glioma (second column), and one healthy brain (third column). (**A**,**B**,**C**) Voxel localization on fluid attenuated inversion recovery (FLAIR) images; (**D**,**E**,**F**) evaluation of the secondary 2HG-peak at 4.02 ppm with the “edited” Mescher–Garwood point-resolved spectroscopy (MEGA-PRESS) sequence; (**G**,**H**,**I**) evaluation of the primary 2HG-peak at 2.25 ppm with the optimized PRESS sequence. Reproduced with permission from Branzoli et al. [48].

However, MRS performance for 2HG-detection has been proven to depend on tumor cellularity [42] and volume [46]. In addition, 2HG-MRS is a challenging technique to implement, mostly due to a confounding spectral overlap between 2HG and several other metabolites [47]. A possible solution may be represented by an “edited” MEGA-PRESS (Mescher–Garwood point-resolved spectroscopy) 2HG-MRS sequence, recently proposed [48] for the measurement of a secondary 2HG-peak at 4.02 ppm (Figure 2). The secondary peak exhibits lower intensity but less degree of overlap, with very promising results on the initial cohort of 24 LGG (sensitivity/specificity 1.0/1.0). A subsequent prospective study involving 57 gliomas (both LGG and GBM) [49] discussed potential optimal cutoffs for 2HG-peak, and confirmed the high diagnostic accuracy of MEGA-PRESS 2HG-MRS (sensitivity/specificity/AUC ranging 0.76–0.80/0.76–1.0/0.79–0.89).

Evidence provided by these studies is overall very supportive of a central role of 2HG-MRS for diagnosis, prognosis stratification, and treatment response assessment in the neuro-oncology scenario of the next decade. In particular, MEGA-PRESS seems to be a valid option to overcome the technical obstacles of 2HG-MRS. One last concern deserving further evaluation is raised by the notion of IDH2^mut^ (more common in oligodendrogliomas) being associated with a greater 2HG accumulation than IDH1^mut^ (more common in astrocytomas), which has a significant impact on 2HG-MRS measurements [50] and may have relevant implications when selecting cutoffs for diagnosis.

## 4. 1p/19q Codeletion Determination for Oligodendrogliomas

Oligodendrogliomas are oligodendrocyte-derived tumors, whose current diagnosis requires the demonstration of IDH^mut^1p19q^cod^ molecular status [1], a molecular marker associated with better prognosis and treatment response [51]. Some cMRI findings [52] may direct toward the hypothesis of oligodendroglioma, involving, first of all, the presence of two peculiar features: calcifications and cortical–subcortical involvement. Furthermore, oligodendrogliomas often exhibit an inhomogeneous signal, may present cystic or hemorrhagic areas, and show absent/minimal contrast enhancement (patchy or dot-like, when present). On aMRI, IDH^mut^1p19q^cod^ represents an important exception to the aforementioned “rule of thumb”, as they are characterized by intermediate diffusion and perfusion/permeability features between IDH^wt^ and IDH^mut^1p19q^int^ despite their better prognosis, as demonstrated by several studies [10,11,25,27,28,29]. More in detail, oligodendrogliomas exhibit lower ADC and higher perfusion/permeability markers compared to diffuse astrocytomas, and such aMRI markers do not vary between grade 2 and 3 IDH^mut^1p19q^cod^ [10,11]. These findings, which are counterintuitive considering their mild prognosis, have been attributed to some peculiar microscopic features of oligodendrogliomas, which seem appreciable even in the lower grades. In fact, intralesional calcifications and a relatively high cellularity, with cells showing a cluster growth pattern, may be responsible for decreased ADC-values [53]. On the other hand, their flourishing microvascular networks (“chicken-wire” networks) and the cortical involvement account for higher perfusion/permeability metrics [10]. This mismatch between aMRI features and prognosis in IDH^mut^1p19q^cod^ can be misleading, as their aMRI behavior may mimic higher-grade gliomas (HGG), as well as some cMRI findings such as contrast enhancement and relatively indistinct margins [27].

A recent study [53] applied ADC and rCBV histogram metrics to 71 LGG and confirmed that IDH^mut^1p19q^cod^-LGG exhibit aMRI characteristics that are compatible with a higher cellularity (ADC_mean_), microvascularity (rCBF_mean_), and vascular heterogeneity (rCBV_peak_) than IDH^mut^1p19q^int^-LGG, and it reported a good diagnostic performance of the combination of such parameters for the detection of 1p19q^cod^-status (sensitivity/specificity/AUC 0.92/0.81/0.84). Despite their relevance, the impact of these results is limited by the fact that the cohort did not include IDH^wt^-gliomas, which present with more aggressive aMRI-metrics, often partially overlapping with oligodendrogliomas.

Similarly to IDH-profiling, an innovative approach for 1p19q-determination could be provided by MRS. In fact, the loss of two enzymes located on chromosome-1p results in a cystathionine accumulation, which can be measured by MRS after the subtraction of the spectra obtained from an edit-on and an edit-off condition [54]. Cystathionine level on MRS was proven significantly higher in IDH^mut^1p19q^cod^, designating this metabolite as a potential marker for 1p19q^cod^, in case its diagnostic reliability will be assessed.

## 5. Additional Molecular Markers in GBM: Epidermal Growth Factor Receptor (EGFR) Modifications and O^6^-Methylguanine DNA Methyltransferase (MGMT) Methylation

In addition to IDH-status, other molecular features hold a prognostic significance in GBM, including EGFR modifications (mainly mutations or amplifications) and MGMT methylation.

Epithelial growth factor receptor (EGFR) is altered in ~60% of de novo GBM and ~10% of secondary GBM [55]; its most common alteration (~33% of GBM) is EGFR variant-III (EGFvIII), presenting a mutation in the extracellular portion [56]. EGFR modifications are believed to have a role in tumor invasiveness (regulating proliferation and motility of tumor cells) and often occur in the infiltrating periphery of GBM, accounting for the fuzzier appearance of tumor margins [55]. Despite not representing a diagnostic criterion for WHO 2016 GBM classification, the identification of EGFR modifications (and specifically EGFRvIII) may have prognostic and therapeutic implications (even though the exact prognostic meaning of EGFRvIII is still controversial) [57,58,59]. EGFR alterations have been associated with lower ADC [60], higher rCBV, and lower PSR (percentage signal recovery, a DSC-derived metric reflecting contrast leakage) [61], as well as higher rV_p_ and rK^trans^ [62]. Considering these aMRI markers alone, across studies, rVp-derived histogram metrics (mean, 90th percentile, 70th percentile) were the most accurate (AUC ~0.82) [62]. A recent study [63] on 129 GBM patients (discovery/replication cohort: 75/54) developed a multiparametric model (featuring DSC, ADC, DTI, and cMRI histogram analysis from tumor VOI) capable of predicting EGFRvIII (sensitivity/specificity/AUC 0.83–0.79/0.86–0.90/0.85–0.86 for the two cohorts, respectively). Notably, this aMRI analysis revealed a complex imaging signature of EGFRvIII-GBM that exhibits higher-rCBV and lower-ADC both in the enhancing tissue and in nonenhancing peripheral tissue, but also contains peculiar low-rCBV (hypovascular “prenecrotic”) foci within the enhancing tissue. In another study [56] on 142 GBM patients (discovery/replication cohort: 64/78), two manual ROIs were placed in nonenhancing T2/FLAIR alteration areas, respectively adjacent to the enhancing tissue and distant from it. In EGFRvIII-GBM, the high peripheral vascularization caused the “near”-ROI and the “far”-ROI to display very similar DSC curves, whereas, in non-EGFRvIII-GBM, the curves diverged (the “near”-ROI exhibiting higher vascularity). An index of separability between the two ROIs was obtained (by representing the DSC curve vectors in a principal component analysis), which showed high diagnostic performance for EGFRvIII-prediction (AUC 0.88). In a representative case illustrated by the authors, the between-ROI difference was also appreciable with DSC curve qualitative visualization. Notably, including DTI- and cMRI-derived measures in the model did not significantly improve the diagnostic performance.

The methylation of the O^6^-methylguanine DNA methyltransferase promoter (MGMT methylation) is an epigenetic modification that reduces the expression of MGMT, a DNA-repair enzyme. MGMT methylation in GBM is a positive prognostic factor for treatment response (with temozolomide and radiation-therapy), and it is also correlated to a better prognosis regardless of treatment [64].

Multiple studies attempted to discriminate MGMT status on the basis of ADC values, with very inconclusive results. In fact, some authors reported ADC measures being higher in MGMT-methylated [65,66,67], others lower [68], and others found no between-group difference [69]. Some authors [70] even found diverging results in the same GBM cohort, with ADC_min_ being lower or higher depending upon the different approach for extracting ADC_min_ (either an absolute minimum value or a “two-mixture distribution” histogram analysis, respectively). This result was consistent with diverging results from previous studies employing these two methods [65,68], suggesting that the different approach may partially account for these controversial findings.

In one study [69], DCE and DTI metrics were extracted from a VOI of the enhancing tumor, and K^trans^ exhibited significantly higher values in MGMT-methylated GBM (sensitivity/specificity/AUC 0.56/0.85/0.76), whereas other aMRI metrics and cMRI features did not differ between groups. K^trans^ being higher in MGMT-methylated GBM is surprising given that K^trans^ is generally considered a marker of immature and “leaking” neovascular structures, thus correlating with more aggressive tumors. It has been speculated that endothelial permeability in MGMT-methylated GBM could be related to a better temozolomide penetration in the tumor [69]. This hypothesis would be consistent with the notion of elevated K^trans^ being correlated with better prognosis [71] and with MGMT methylation being associated with pseudoprogression [72,73], a post-treatment phenomenon representing a positive prognostic factor [74,75] and supposedly reflecting edematous and permeability alterations linked to treatment effectiveness [76].

## 6. Novel GBM-Defining Genotypes: EGFR Amplification and Telomerase Reverse Transcriptase (TERT) Mutation in IDH^wt^-Gliomas

In light of the cIMPACT-NOW Update 6 [77], the diagnostic criteria for GBM have been further revised toward an even higher centrality of molecular profiling. On one hand, the classic histopathological hallmarks (microvascular proliferation and necrosis) are currently not sufficient for a diagnosis of GBM, as the absence of IDH^wt^-status defines a novel subtype named IDH^mut^-astrocytoma grade 4. Conversely, the presence of peculiar molecular features (IDH^wt^-status associated with EGFR amplification, TERT mutation, or +7/−10 chromosome copy number changes) suffices for the definition of GBM, regardless of microscopical features (that is, even though the microscopical evaluation advocates for LGG). This is a rather recent definition, and only few studies addressed the identification of these novel GBM-defining genotypes within IDH^wt^-LGG.

A retrospective qualitative evaluation on 71 LGG [78] reported EGFR amplification being almost exclusively seen in IDH^wt^-LGG and significantly correlating with mild (not avid) contrast enhancement, with >5% enhancing tumor, and with infiltrative/mixed growth pattern. In addition, diffusion restriction was rare in this cohort, but exclusively seen in EGFR-amplified IDH^wt^-LGG. Park and colleagues [79] extracted VOI-derived ADC, DTI, DSC, and DCE parameters from 49 IDH^wt^-LGG, and identified lower ADC_mean_ as an independent predictor of EGFR amplification (AUC 0.75) and perfusion/permeability parameters (nCBF_mean_, nCBV_mean_, and V_p-mean_, where “n” stands for “normalized”) as independent predictors of TERT mutation (AUC 0.85 for V_p-mean_). These results are particularly promising, suggesting that aMRI may play a role in the in-vivo detection of these novel GBM markers, not only aimed at an optimized diagnosis and risk stratification, but also potentially at therapy guidance, as TERT- and EGFR-targeted therapy [80,81] have been explored for gliomas.

## 7. Diffuse Midline Gliomas H3K27M-Mutated

**Figure 3:**
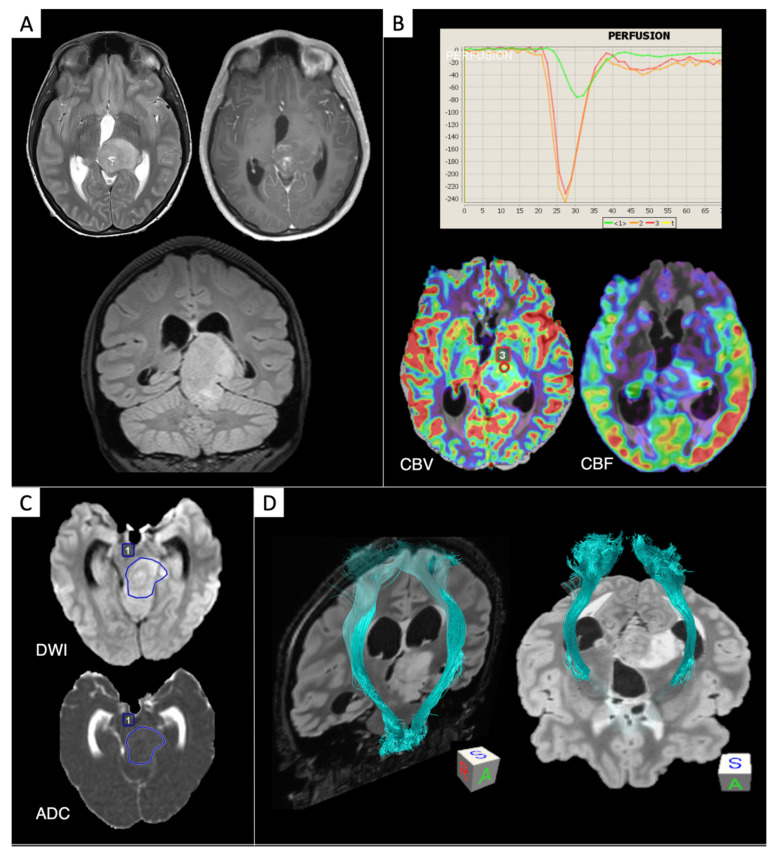
Diffuse midline glioma H3K27M^mut^. MRI datasets were retrieved from the archive of our Institution. (**A**) Conventional MRI (cMRI): T2, contrast-enhanced T1, FLAIR (coronal section). (**B**) PWI: dynamic susceptibility contrast (DSC) curve (red and orange: tumor ROIs; green: NAWM), DSC-cerebral blood volume (CBV) (red: tumor ROI), arterial spin labeling (ASL)-cerebral blood flow (CBF). DSC “hotspot” ROI evaluation revealed “aggressive” perfusion features (red ROI: CBV_max_, 4 mL/100 g; CBV_max_/CBV_NAWM_, 6.48); ASL-CBF showed highly perfused spots within the tumor tissue. (**C**) dMRI: a clinically feasible single-slice ROI evaluation revealed low diffusion parameters (blue ROI: ADC_mean_, 0.87 mm^2^/s; ADC_mean_/ADC_NAWM_ ratio, 1.19). (**D**) Q-ball tractography of corticospinal tracts, exhibiting a mild ventrolateral dislocation of the left tract due to the mass effect of the lesion.

DMG-H3K27M^mut^ are diffuse gliomas arising on the midline (Figure 3), often infra-tentorial or thalamic and mostly seen in the pediatric population [1], and they include the previously defined diffuse intrinsic pontine gliomas (DIPGs). DMG-H3K27M^mut^ diagnosis requires the identification of K27M^mut^ of H3-histone, resulting in tumorigenesis through a reduced activity of H3, modulating chromatin changes and gene expression [4]. The identification of H3K27M^mut^ status is particularly relevant, since DMG-H3K27M^mut^ are considered WHO grade 4 tumors, regardless of microscopic appearance, due to their poor prognosis [4]. Features on cMRI may be variable and nondistinctive [82,83], exhibiting mainly absent or partial/peripheral enhancement, frequently a solid appearance, and rarely hemorrhagic areas.

Different studies achieved discordant results for H3K27M status determination through aMRI quantitative analyses. Piccardo et al. [84] reported higher rCBV_max_, lower rADC_min_ (both determined through manual ROIs with a hotspot approach), and higher choline/creatine ratio of DMG-H3K27M^mut^ compared to H3K27M^wt^-gliomas arising from the midline (AUC 0.85/0.81/0.78 for the three metrics, respectively). Another article [85] proposed ADC measures extracted from tumoral and peritumoral manual ROIs for H3K27M^mut^-prediction, with good diagnostic performance (AUC: ranging from 0.81–0.87 for single-ROIs; 0.87 combining tumoral and peritumoral rADC_min_). However, other authors [83,86] found no association between low ADC-values and H3K27M^mut^, and a recent meta-analysis [87] reported that DMG-H3K27M^mut^ generally exhibit similar aMRI features (ADC and ASL-CBF) to LGG rather than HGG and, therefore, are often distinguishable from medulloblastomas but not from pilocytic astrocytomas. Similarly, findings from animal models suggest that H3K27M status does not affect tumor permeability, as no association with K^trans^-variations was found [88]. The inconsistency between studies may partially arise from the heterogeneity in the midline H3K27M^wt^-gliomas enrolled, which may include both HGG and LGG. Future studies should perhaps focus on the differentiation with midline H3K27M^wt^-LGG, as it determines a major prognostic difference.

On the other hand, there is robust evidence that ADC correlates with survival in DIPG, regardless of H3K27M-status [83,89,90], once again suggesting that dMRI may have an independent prognostic value, as it reflects tumor biological features as cellular density and extracellular matrix alterations.

According to cIMPACT-NOW Update 6 [77], another mutation in H3-histone (H3.3-G34^mut^) has been proposed as a distinctive molecular marker of a novel tumor type, diffuse glioma H3.3-G34^mut^, a pediatric cerebral hemispheric glioma bearing a similar prognosis as DMG-H3K27M^mut^ [91,92]. Recent studies provided a description of some H3.3-G34^mut^ cMRI features [92], including inhomogeneous appearance, scarce/absent contrast enhancement, and sometimes cystic components, but aMRI features of this new tumor type are still to be assessed.

## 8. Medulloblastomas

The molecular classification of medulloblastomas (grade 4) currently acknowledges four subtypes: WNT-activated, SHH-activated, and non-WNT/non-SHH subgroups including “group 3” and “group 4” [1]. For medulloblastomas, molecular categories also correspond to different prognoses, which vary from good (WNT-activated) to intermediate (SHH-activated and group 4) to poor (group 3) [3]. Some cMRI characteristics may aid in the subtype differentiation [93]: hemispheric localizations are exclusively seen in SHH-activated, the absence of contrast-uptake is mostly described in group 4, and intratumoral bleeding is thought to be more frequent in WNT-activated.

Few studies focused on the application of aMRI for the subtype identification of medulloblastomas.

A recent study assessed the capability of MRS to distinguish medulloblastoma subtypes [94] and found group 3/4 to reveal distinct spectral features when compared to SHH-activated, including differences in taurine peaks (detectable in the former and absent in the latter) and in lipid peaks (more prominent in the latter). These authors proposed a model based on the measurements of five metabolites (taurine, lipid-13a, myo-inositol, creatine, and aspartate) with very good diagnostic performance (AUC 0.88).

Other researchers systematically compared ROI-derived ADC-values from medulloblastomas in 93 pediatric patients [95], and they notably reported higher ADC-values for group 3/4 and lower for WNT-activated, supposedly due to the anaplastic variant being more represented for group 3/4 in their cohort and to a higher cellularity in WNT-activated subtypes. However, ADC-values largely overlapped among tumor subtypes. On the other hand, ADC histogram metrics (entropy and 90th percentile) were proven to correlate with the histological proliferation marker Ki67, suggesting a potential role of ADC as an independent prognostic factor in medulloblastomas [96]. In addition, various ROI-derived and histogram-derived ADC-metrics were proven useful for the differential diagnosis between medulloblastoma and other infra-tentorial neoplasms such as pilocytic astrocytomas, metastases, hemangioblastomas, and ependymomas [97,98,99]. Moreover, DTI histogram metrics showed different values in medulloblastomas and pilocytic astrocytomas [100], with mean diffusivity (MD) outperforming other DTI-metrics, consistently with the notion of MD providing insights analogous to ADC regarding tumor microstructure. Lastly, a recent meta-analysis [87], based on 14 studies, evaluated ROI-derived ADC and ASL-CBF extracted from pediatric brain tumors with a “hotspot” approach and reported high accuracy (ADC_min_ = 0.97; nCBF_max_ = 0.83) in distinguishing low-grade lesions from high-grade lesions (including medulloblastomas, fairly represented in their cohort).

## 9. DTI and DKI for Glioma Assessment

DTI is a dMRI technique that evaluates the orientation of water diffusion in biologic tissues as a result of constraints represented by oriented cell membranes and myelin [15], and it provides a number of metrics, including *p* (pure isotropic diffusion, reflecting diffusion lacking a preferential direction), *q* (pure anisotropic diffusion, reflecting diffusion with a preferential direction), FA (fractional anisotropy, reflecting the degree of anisotropic diffusion in a single direction compared to other directions), and MD. Despite its primary function in neuro-oncology being to provide datasets to perform tractography, depicting the trajectory of eloquent white-matter tracts in the presurgical brain mapping [101,102], multiple studies advocate for a role of DTI in delineating tumor margins and in characterizing tumor tissue. In gliomas, altered DTI-metrics showed high sensitivity in detecting tumor infiltration beyond T2-signal alterations [103], and the correct identification of tumor extension has implications in the planning of surgery and radiation treatment [10,104]. In a recent work [103], *p* and *q* were employed to assess the volume of peripheral infiltration in LGG, and they demonstrated a markedly increased infiltration in IDH^wt^-LGG compared to IDH^mut^-LGG (with no difference for 1p19q^cod^-status), and the authors advocated for decreased *p* and increased *q* in tumor-infiltrated areas reflecting a higher cell density in the presence of intact axons. Other studies evaluated the diagnostic performance of DTI-metrics for IDH-status determination, as higher FA-values are seen in IDH^wt^-gliomas [105,106], potentially reflecting either cell atypia [106] or microvascular structures [105,107]. One study [105] compared ADC- and FA-derived measures, obtained with a “hotspot” manual ROI approach (after the visual identification of low-ADC and high-FA areas), and it reported similar capability of IDH-prediction (AUC ranging 0.76–0.94) for LGG, whereas only ADC (not FA) predicted IDH-status in GBM (AUC 0.66–0.70). Overall, the authors suggested employing rADC_min_ rather than FA measures. Aliotta and colleagues [106] confirmed a similar performance between ADC and FA for IDH-prediction, in a study employing histogram metrics and few texture features, but demonstrated an improved diagnostic performance using an ADC + FA combination model (sensitivity/specificity/AUC 0.80/0.80/0.90). Interestingly, these authors also tested an estimated FA obtained from accelerated scans (three directions), which also increased IDH-prediction accuracy compared to ADC alone (with slightly worse results than classic FA). Other authors [108] obtained higher accuracy (AUC 0.92–0.95) for distinguishing IDH^wt^- and IDH^mut^-LGG through a machine-learning model based on FA and B0 texture features.

While ADC and DTI assume water displacement following a Gaussian distribution, DKI is a dMRI technique that measures the degree of directional non-Gaussian diffusion [109], better representing the restricted component of diffusion in biological tissue [110]. DKI-derived parameters reflect non-Gaussian diffusion along the principal orientation (Ka, axial kurtosis), the secondary orientations (Kr, radial kurtosis), and as a mean of the three directions (MK, mean kurtosis).

DKI has been widely applied to grade differentiation in gliomas, and a recent meta-analysis [108] reported a good diagnostic performance of MK (across 12 studies, pooled sensitivity/specificity/AUC 0.87/0.85/0.92) in distinguishing LGG from HGG, using a cutoff value ranging from 0.5 to 0.6 across studies. As for IDH-determination, Ka seems to be the most promising marker, according to two studies [110,111]. In the first [111], in a cohort of 52 gliomas, Ka extracted from manual ROIs outperformed DTI-metrics and other DKI-metrics in identifying IDH^wt^-status (with sensitivity/specificity/AUC 0.75/0.74/0.72). In addition, DKI-metrics (positively) and MD (negatively) were correlated to Ki67 proliferation marker, whereas FA was not, suggesting that the measurement of non-Gaussian diffusion may better reflect tumor cellularity. In the second [110], IDH^wt^-status in 66 gliomas was predicted through a combined model employing DTI- and DKI-metrics, as well as clinical information, and Ka was the most important parameter for IDH-prediction. Furthermore, metric extraction from multiple manual ROIs did not affect the accuracy of the model (as opposed to whole-tumor VOI extraction), and actually the ROI-based model performed better in their cohort (AUC 0.85 vs. 0.77). The authors speculated that Ka may reflect axonal integrity and density, thus being lower in the case of infiltrative tumor growth [110,112].

As for oligodendrogliomas (IDH^mut^1p19q^cod^), similarly to ADC and perfusion/permeability metrics, this tumor type also seems to be particularly challenging to identify for DTI and DKI analysis. In fact, IDH^mut^1p19q^cod^-LGG exhibit intermediate FA compared to IDH^mut^1p19q^int^- and IDH^wt^-gliomas [106], and DKI-metrics do not significantly vary between IDH^mut^1p19q^int^ and IDH^mut^1p19q^del^ [110].

## 10. Biophysical Models: Toward Microstructural dMRI

Novel dMRI approaches are being developed, based on “biophysical models”, which assume a certain underlying tissue microstructure defined a priori, and which are organized in multiple water compartments [17]. These techniques provide unprecedented insights into tissue biology, potentially providing additional information for tumor characterization. Neurite orientation dispersion and density imaging (NODDI) is a relatively new model segregating the diffusion signal from intraneurite (FICV, intracellular volume fraction), extraneurite (FECV, extracellular volume fraction), and free water (FISO, fraction of isotropic Gaussian diffusion) compartments [112,113]. In their study on 192 glioma patients, Figini et al. [114] compared the ability of predicting IDH status of NODDI-, DTI-, and DKI-metrics extracted from single-plane ROIs. In their cohort, NODDI had no advantages over the remaining techniques, with similar diagnostic performance (AUC ranging 0.72–0.76) when distinguishing IDH^wt^- from IDH^mut^-LGG, whereas kurtosis anisotropy was the only metric displaying different values in IDH^wt^- and in IDH^mut^-GBM. Despite these findings not seeming to advocate for a dramatic contribution of NODDI in the assessment of brain tumors, it should be pointed out that this model might not be the most adequate for tumor characterization, due to its a priori constraints that were set in order to represent normal brain tissue rather than neoplastic tissue. Indeed, some authors already reported on NODDI being inaccurate when applied to certain pathological conditions, particularly in the case of gliomas, where neurite density contrast should not be interpreted as due to neurites [115]. However, a possible application of NODDI in neuro-oncology could lie in the evaluation of the peritumoral tissue. A recent study [116] demonstrated that quantitative NODDI-metrics extracted along the trajectory of white-matter peritumoral tracts are more sensitive than DTI-metrics in detecting white-matter microstructural alterations and contribute to characterizing infiltrative and/or vasogenic edema. The role of NODDI in the distinction between infiltrative and vasogenic edema was also previously suggested by other authors, either through a visual inspection of NODDI color maps [117] or through the extraction of quantitative NODDI metrics [118]. Indeed, Kadota et al. [118] reported a very good diagnostic performance (AUC 0.87) of the peritumoral FECV in differentiating GBM and solitary metastasis, supporting the hypothesis of infiltrative edema being characterized by a higher FECV than vasogenic edema, as also previously suggested by other studies [115]. Unlike NODDI, other biophysical models—such as vascular, extracellular, and restricted diffusion for cytometry in tumors (VERDICT)—were specifically designed to represent neoplastic tissue. VERDICT [119] assumes an anisotropic vascular compartment, an extracellular compartment with isotropic hindered diffusion, and an intracellular compartment with restricted diffusion, and it also estimates cell radius. Originally optimized for prostate neoplasms, it was recently applied to gliomas [120]. A recent study [120] employed a clinically feasible VERDICT sequence (5 min 30 s) and compared VERDICT-metrics in IDH^mut^-LGG and IDH^wt^-HGG. A significant difference in the “intracellular compartment” was found between groups, with this metric being higher in IDH^wt^-HGG, whereas no significant ADC differences were seen in this cohort. No between-group differences were found in cell radius from histopathology nor VERDICT, whereas another study [121] described a difference in VERDICT radius between GBM and LGG. Despite further studies potentially validating a hypothetical role of cell radius in diagnosis, the major potential application of this metric could be in the treatment response assessment, moving from the notion of cell shrinkage being an early marker of tumor cell death, as already demonstrated by applying VERDICT to animal models [121]. Lastly, while the majority of biophysical models postulate water compartments to be impermeable, filter-exchange imaging (FEXI) is a novel technique that disentangles the signal effects ascribable to water exchange across membranes and to restricted diffusion, thereby estimating an apparent exchange rate (AXR) between water compartments [122,123]. In a recent article, Lampinen et al. [124] employed FEXI to demonstrate differences in water exchange between astrocytomas and meningiomas, suggesting that AXR could provide novel insights into the degree of cell membrane permeability, which may vary between different tumor types.

## 11. BOLD Imaging to Evaluate Tumor Microvascularization and Oxygen Metabolism

Blood-oxygen-level-dependent imaging (BOLD) is an aMRI technique exploiting the paramagnetic properties of deoxyhemoglobin to infer data regarding blood oxygenation [125]. In the presurgical workup of brain tumors, this property is applied in functional-MRI (fMRI) studies aimed at identifying eloquent cortical areas to be spared during the surgical resection [126,127,128]. While fMRI is based on the assumption that neural activation is coupled with a hemodynamic response (neurovascular coupling) in the healthy gray matter, tumor-infiltrated tissue may exhibit neurovascular uncoupling due to an impaired cerebrovascular reactivity, and BOLD itself can be employed to detect areas of uncoupling [129].

Another study [130] further proved that quantitative BOLD is capable of providing useful insights regarding tumor-induced vascular abnormalities, in particular detecting areas of tumor-like BOLD alterations beyond cMRI-defined tumor margins. The authors proposed BOLD alterations as a marker to reveal tumor-infiltrated tissue and to detect post-surgical residual disease. In addition, they reported IDH^mut^-gliomas harboring R132H-mutation (R132H^+^-IDH^mut^, a mutation associated with better prognosis) exhibiting a remarkably inferior BOLD alteration extension (measured by means of an index named “BOLD-only fraction” of the tumor—BOF) than R132H^−^-gliomas. BOF had an excellent diagnostic accuracy in discriminating R132H-status (AUC 0.98) in this cohort of 39 diffuse gliomas, advocating for a role of BOLD in molecular profiling.

**Figure 4:**
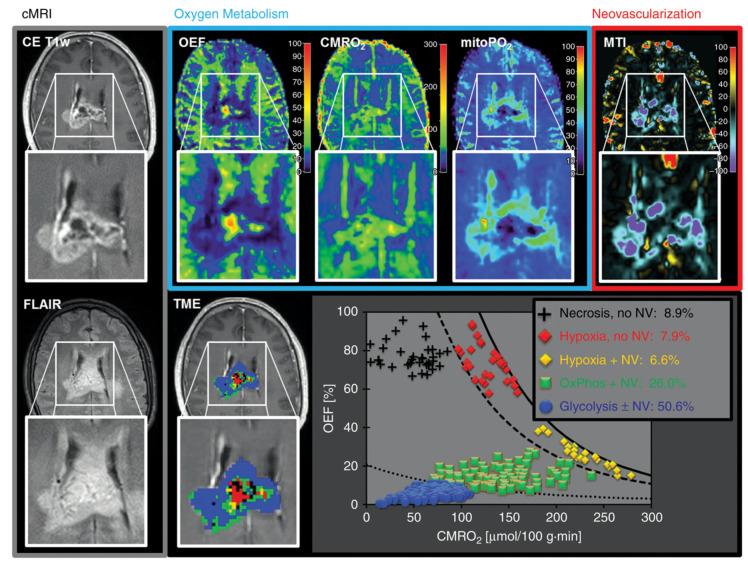
Clustering of tumor microenvironments (TMEs) in a IDH1^wt^-glioblastoma (GBM). Maps representing advanced MRI (aMRI) markers of oxygen metabolism and neovascularization were calculated (upper row), and specific TMEs were identified on the basis of reciprocal variations of aMRI markers voxel-wise (lower row: TME map and scatter plot). According to glycolysis, hypoxia, and neovasculature markers, this GBM was classified as showing a “glycolytic-dominated” phenotype with functional neovasculature, a phenotype associated with better prognosis. Reproduced with permission from Stadlbauer et al. [132].

Stadlbauer and colleagues [131,132,133] employed a novel multiparametric approach—combining DSC, BOLD datasets, and ADC—to assess tumor oxygen metabolism and characterize microvascularization by estimating a number of insightful quantitative metrics including oxygen extraction fraction (OEF), cerebral metabolic rate of oxygen (CMRO_2_), radius (R_U_) and density (N_U_) of microvessels, microvessel type indicator (MTI, distinguishing between predominantly arteriolar and predominantly venular microvasculature), micro- and macrovascular transit time heterogeneity (VTH), and mitochondrial oxygen tension (mitoPO_2_). LGG exhibit higher OEF and lower CMRO_2_, while HGG showed lower OEF, higher CMRO_2_, and higher neovascularization markers (N_U_, R_U_, MTI, CBV); these findings were interpreted as LGG requiring an increased oxygen extraction (OEF) to meet the increased metabolic needs in the absence of neovascularization, while, in HGG, the additional oxygen demand (CMRO_2_) drives neoangiogenesis, supplying oxygen without a higher extraction [131]. In addition, the authors reported a good diagnostic performance of these metrics for IDH-prediction (AUC: 0.90 for MTI in all grades; 0.85 for MTI in HGG; 0.82 for CMRO_2_ in LGG). In another study by the same research group [132], the reciprocal variations of these metrics were employed to characterize six main tumor microenvironments in IDH^wt^-GBM: necrosis, hypoxia with defective neovasculature, hypoxia with functional neovasculature, normoxia with functional neovasculature, glycolysis without neovasculature, and glycolysis with functional neovasculature. Two main tumor types were identified depending on the degree of representation of “glycolysis with functional neovasculature” (G + NV) microenvironment: a “glycolytic-dominated” phenotype with functional neovasculature (G + NV ranging 34–86% of tumor volume, Figure 4), and a “necrotic/hypoxic-dominated” phenotype with defective neovasculature (G + NV 0.9–18%). These two tumor types exhibited a significant difference in progression-free survival, confirming that aMRI markers (in this case reflecting neoangiogenesis and oxygen-metabolism) may represent prognostic factors independent from molecular profiles (the study included only IDH^wt^-GBM). As a confirmation of the potential role of BOLD for molecular profiling and prognosis stratification, other authors [134] reported rOEF and R_2′_ (a BOLD-metric reflecting vascular oxygen-saturation) as being capable of discerning EGFR-amplification in gliomas (AUC 0.70 and 0.72, respectively), and both metrics were significant predictors of overall survival.

## 12. Frontiers of Ultra-High-Field Imaging

MRI field strengths of 7 Tesla (7T) or more, namely, “ultra-high-field”, are characterized by peculiar physical characteristics, including enhanced susceptibility and chemical shift effects, T1 relaxation time modifications, and a higher signal-to-noise ratio (SNR) [135].

Susceptibility-based techniques such as susceptibility-weighted imaging (SWI), quantitative susceptibility mapping (QSM), and BOLD are more sensitive at ultra-high-field, and their application to brain tumors may aid the evaluation of neovasculature, the distinction between microhemorrhage and calcifications (by means of QSM, with potential clinical relevance for differential diagnosis between GBM and oligodendrogliomas), and the presurgical brain mapping with fMRI [135,136,137].

One technique that benefits from enhanced chemical shift effects is chemical exchange saturation transfer (CEST), whose contrast reflects the exchange between free protons and bound protons, mostly depending on protein concentration in the biological tissue and other microenvironmental factors [138]. CEST imaging provides novel insights into the tumor microenvironment, with potential diagnostic applications that are yet to be fully explored. A recent study [138], for instance, reported histogram-based CEST-metrics as excellent predictors of IDH-status in gliomas (with AUC as high as 0.98, depending on the metric). Furthermore, one peculiar application of CEST imaging is the imaging of injected glucose (glucoCEST and dynamic glucose-enhanced imaging—DGE) to assess its uptake in the neoplastic tissue [139,140], potentially representing a future alternative to positron emission tomography (PET).

The increase in T1 relaxation time, on the other hand, is a favorable condition not only for time-of-flight (TOF) angiography, but also for ASL, thus suggesting that ultra-high-field may offer additional advantages for the evaluation of tumor perfusion [135,141].

A higher SNR not only results in a spatial-resolution improvement, but also allows for a better sensitivity for the detection of metabolite peaks in MRS, and it enables for imaging based on nuclei other than hydrogen (X-nuclei) [135,137], including sodium-23 (^23^Na) [142], chlorine-35 (^35^Cl) [143], potassium-39 (^39^K) [144], and oxygen-17 (^17^O) [145]. While the imaging of some X-nuclei (e.g., ^23^Na) has also been proven feasible at 3 T [146] (but still greatly benefits from ultra-high-field), other X-nuclei have been only imaged at 7 T. X-nuclei MRI has the potential to provide unprecedented information regarding tissue electrolyte homeostasis, which is yet to be explored. The ^23^Na signal, for instance, probes tissue viability, and was found increased in tumors due to membrane depolarization in the cell division initiating phase (intracellular-^23^Na) and to extracellular space expansion (extracellular-^23^Na) [137]. Nagel and colleagues reported total ^23^Na being elevated in all tumors, while the relaxation-weighted ^23^Na signal was specifically increased in GBM [142], and they proposed a ^23^Na inversion-recovery (IR) sequence that selectively images intracellular-^23^Na [143]. Overall, ^23^Na-MRI shows a potential application for neoplastic tissue characterization, and ^23^Na-IR may aid the distinction between infiltration (intracellular ^23^Na-elevation) and edema (extracellular ^23^Na-elevation) in the peritumoral tissue. Lastly, ^17^O-MRI gives the unique opportunity to directly measure oxygen metabolism (CMRO_2_) in brain tumors after the patient inhales ^17^O, which is used as a tracer, being very rare in the natural atmosphere [145].

## 13. Contributions from Artificial Intelligence

In recent years, machine learning (ML) has been applied to neuroimaging analyses for a number of applications, including tumor segmentation, a mandatory step for all radiomic studies willing to assess quantitative features from whole-tumor VOIs. Segmentation consists of obtaining 3D VOIs of the tumor from cMRI images, and it is traditionally performed manually by trained personnel. Tumor VOIs can then be registered to other cMRI or aMRI datasets in order to extract quantitative values, including histogram metrics and texture features. However, the need for human intervention comes at great cost; the segmentation procedure is highly time-consuming, requires training, and is ultimately operator-dependent [147]. To address these limitations, deep learning (DL) methods through convolutional neural networks (CNNs) have been proposed to perform automated tumor segmentation [148]. While “classic” ML requires “manually engineered” features as inputs, DL approaches autonomously learn to select useful features for the task, thereby bypassing the need for human intervention [149]. Extensive literature regarding possible strategies for DL segmentation has been produced [148,149,150,151,152], which already reached very satisfactory results in the most recent studies [148], and models showing good performance are already available open-access (e.g., https://github.com/NeuroAI-HD/HD-GLIO) [153,154]. Automated segmentation will make the extraction of quantitative MRI-metrics dramatically easier, less time-consuming, and more consistent across operators and institutions. In addition, if implemented on clinical picture archiving and documentation systems (PACS), it would enable a more accurate evaluation of lesional burden at diagnosis and residual disease after surgery.

Multiple studies explored the value of DL approaches for predicting prognosis, tumor grades, and molecular profiles, and for distinguishing progressive-disease from pseudoprogression after treatment [155]. In a recent study on 259 patients, Chang et al. [156] employed a CNN model based on cMRI datasets for the molecular profiling of both LGG and HGG, and they reported a very high accuracy for the prediction of IDH-, 1p19q-, and MGMT-status (0.94/0.92/0.83, respectively). Another study [157] identified an AI-computed radiomic signature that successfully stratified GBM patients in low and high risk, with a significant difference in overall survival, and including clinical data in the model allowed for an even better stratification. Verma and colleagues [158] also adopted an AI model to predict the prognosis of treatment-naïve GBM on the basis of cMRI-extracted advanced radiomic features. Furthermore, these authors assessed the correspondence between radiomic features and histopathologic findings in order to characterize tumor tissue microenvironment. Their analyses revealed how radiomics heterogeneity markers reflect tumor heterogeneity in the high-risk GBM, corresponding to a heterogeneous microenvironment of the hypoxic niche (in the peri-necrotic area) and to a tumor niche enriched in hyperplastic blood vessels.

## 14. Conclusions

In the current neuro-oncology scenario, tumor classifications are constantly updated in order to match the latest pieces of evidence regarding the different prognosis and treatment response of tumor entities. aMRI quantitative analyses are a noninvasive source of numerous in vivo biomarkers providing unprecedented insights regarding neoplastic tissue biology and pathophysiology, including tumor microstructure, microvasculature, metabolism, and electrolyte homeostasis.

On one hand, extensive literature has confirmed the role of the most “robust” aMRI-metrics (e.g., ADC and CBV) for the evaluation of tumor aggressiveness, for the prediction of specific molecular signatures that are crucial for diagnosis, and, to some degree, even for prognostic stratification. In this regard, several studies have assessed the reliability of hand-drawn ROIs to sample tumoral and peritumoral aMRI-metrics in order to orient the diagnosis toward a specific molecular type. In addition, multimodality evaluation outperforms single-metric approaches for predicting molecular status. Overall, considering that these aMRI-techniques are widely employed throughout neuroradiological centers and that ROI-based measurements are easy to perform on clinical PACS, a multimodality approach assessing quantitative ADC- and CBV-values alongside with cMRI features seems an applicable approach in the current clinical routine.

On the other hand, results from multiple studies advocate for 2HG-MRS assuming an increasingly central role in the next decade not only for the IDH-status assessment at the time of diagnosis (which is fundamental according to the current clinical workup of gliomas), but also for treatment response assessment, specifically for directly evaluating the effects of the IDH-targeted therapy, which represents an encouraging therapeutical option.

Furthermore, the refined characterization of tumoral features and microenvironments enabled by more advanced approaches (e.g., NODDI, VERDICT, tumor-BOLD, ^23^Na-imaging) opens up the possibility of numerous applications, and their reliability for molecular profiling is still to be explored. Interestingly, shreds of evidence suggest that aMRI metrics providing such detailed and pioneering insights into tumor pathophysiology may bear prognostic value per se, regardless of the correspondence to specific molecular types. Future studies will address the multiple potential underlying diagnostic/prognostic implications of these novel methods, in order to select which novel techniques will have a role in the future clinical practice.

Lastly, the increasing adoption of AI-based models will not only pave the way for a more accurate diagnostic and prognostic assessment, but also explore the usefulness of higher-order cMRI radiomic features, which may better correlate with microscopic findings, representing an alternative to novel aMRI markers for the characterization of tumor tissue microenvironments.
